# The evolution of headache from childhood to adulthood: a review of the literature

**DOI:** 10.1186/1129-2377-15-15

**Published:** 2014-03-18

**Authors:** Fabio Antonaci, Cristina Voiticovschi-Iosob, Anna Luisia Di Stefano, Federica Galli, Aynur Ozge, Umberto Balottin

**Affiliations:** 1 Headache Center, C. Mondino National Institute of Neurology Foundation, IRCCS, University of Pavia, Pavia, Italy; 2 State Medical and Pharmaceutical University “Nicolae Testemitanu”, Chișinău, Republic of Moldova; 3 University of Pavia, Pavia, Italy; 4 C. Mondino National Institute of Neurology Foundation, IRCCS, Pavia, Italy; 5 Department of Neurology, Mersin University School of Medicine, Mersin, Turkey; 6 Child Neurology and Psychiatry Department, C. Mondino National Institute of Neurology Foundation, IRCCS, University of Pavia, Pavia, Italy

**Keywords:** Migraine, Tension-type headache, Cluster headache, Childhood, Adolescence, Evolution

## Abstract

Headache is one of the most common disorders in childhood, with an estimated 75% of children reporting significant headache by the age of 15 years. Pediatric migraine is the most frequent recurrent headache disorder, occurring in up to 28% of older teenagers. Headaches rank third among the illness-related causes of school absenteeism and result in substantial psychosocial impairment among pediatric patients.

The aim of this study was to clarify the evolution of the clinical features of primary headache in the transition from childhood to adulthood through a review of relevant data available in the PubMed and Google Scholar databases for the period 1988 to July 2013.

The search strategy identified 15 published articles which were considered eligible for inclusion in the analysis (i.e. relevant to the investigation of pediatric headache outcome). All were carried out after the publication of the first version of the International Classification of Headache Disorders (ICHD-I).

The availability of data on the evolution of primary headaches over a period of time is important from both a clinical and a public health perspective. The identification of prognostic factors of the evolution of headache (remission or evolution into another headache form) over time should be an objective of future headache research for the development of prevention strategies. Given that headache is a major factor contributing to school absenteeism and poorer quality of life not only in childhood but also in adolescence, understanding the natural history and the management of the different headache forms is vital for our future.

## Introduction

The first description of the different types and clinical characteristics of headache in children was probably that provided by Vahlquist in 1949 [1]. Headache is one of the most common disorders in childhood, with an estimated 75% of children reporting significant headache by the age of 15 years [2]. Headache is rare before the age of 4, but its prevalence increases throughout childhood, peaking at around 13 years of age in both sexes. Prevalence estimates vary according to age, the definition of headache and the method of data collection used, but as many as 75% of school-age children may experience headache infrequently, while around 10% have recurring headaches [3,4]. Studies using the first International Headache Society criteria (IHS, 1988) found prevalence rates of between 3 and 11% in the general population [5,6].

Migraine is a frequent disease with a prevalence of 3-10% in children and adolescents [7,8]. Epidemiological data on tension-type headache (TTH) in young subjects have become available only in the last decade and suggest prevalence rates of between 10 and 24% [9].

Only about 5-10% of cluster headache (CH) patients developed the disease in adolescence, while onset of CH in childhood is very rare [10]. Over the last few years, there has been a growing interest in the evolution of CH over time [11].

Recurrent primary headaches may have a considerable impact on the quality of life of children and adolescents due to the unpredictability of attacks and the presence of accompanying symptoms such as nausea, vomiting, photo and/or phonophobia [12]. Headache in childhood is associated with several psychopathological factors; evaluated prospectively, children with frequent headache were found to have an increased risk of headache, multiple physical symptoms and psychiatric morbidity in adulthood [3]. Reports have shown an association between headache in childhood and several psychosocial factors, such as maternal depression, depression in childhood, social disadvantage and coming from a family with a history of “painful conditions” [13]. Patients with migraine have been found to show more psychopathological symptoms than healthy controls [5]; TTH patients also had more psychopathology than controls, although the difference was more marked in the area of Internalizing disorders [5].

Headache in children and adolescents can lead to impaired psychosocial functioning in various areas of life including family, leisure time activities, working capacity and productivity at school [14]. In addition, affected youngsters may be at a heightened risk of developing additional physical problems in adulthood, as well as mental difficulties such as anxiety and depression [15]. Headaches rank third among illness-related causes of school absenteeism and result in substantial functional impairment among pediatric patients [16].

These data underline the importance of improving understanding of how headache evolves through to adulthood, how its clinical features change, and whether it is possible to identify prognostic factors that indicate whether a patient’s condition (frequency and duration of attacks and symptomatology) is likely to improve, remain unchanged, worsen, evolve into another type of headache, or disappear.

The aim of this study was to review all published data, available in the PubMed and Google Scholar databases, relating to the evolution of the clinical features of headache during the transition from childhood to adulthood.

## Review

### Methods

We searched the PubMed database for literature published between 1988 and July 2013 on the evolution of clinical features of headache from childhood to adulthood. The search was performed using the following combination of terms: “migraine headache” OR “tension-type headache” OR “cluster headache” OR “trigeminal autonomic cephalalgias” OR “paroxysmal hemicranias” AND “evolution” OR “childhood” OR “adolescence”. We also searched Google Scholar, and cross-referenced recent review articles on childhood headache and migraine. Only articles in English and performed in accordance with the diagnostic criteria of the IHS classification [17] were included.

## Results

The search strategy identified 15 published articles that were considered eligible, i.e. relevant to investigation of the outcome of childhood headache; all were included in the analysis.

### Epidemiology of headache

Several studies have been conducted to define the epidemiology of headache in children [6]. Headache disorders in children and adolescents have a high incidence and prevalence, and are also associated with high costs, both to the individual concerned and to society [18]. The reported prevalence of headache among schoolchildren varies greatly, ranging from 5.9 to 82%, depending on the definitions and inclusion criteria used [1,7,18,19]. The vast majority of headaches in this age group are primary and classified as migraine or TTH. The prevalence of headache increases throughout childhood, peaking at about 11–13 years of age in both sexes [3]. Headache is reported in 3-8% of children at 3 years of age [13,20], in 19.5% at the age of 5, and in 37–51.5% at the age of 7 [8,21-24]. The prevalence of headache in 7–15 year-olds ranges from 26 to 82% [8,23,25]. In the study of headache and migraine prevalence in children and adolescents conducted by Abu-Arafeh et al. [26], the estimated prevalence of headache over periods of between one month and lifetime was found to be 58.4%, while the prevalence of migraine over periods ranging from six months to lifetime was 7.7%. Bille et al. [2] studied headache prevalence according to age and found the following distribution: 5.9-37.7% at preschool age, 39% at 6 years, and 70% at 15 years, while the findings of Stewart et al. [27] indicated the following age-distributed prevalence of migraine: 3% in children between 3–7 years, 4-11% in those aged 7–11 years, and 8-23% in 11–15 year olds. In addition, the median age at onset was estimated to be 7.2 years in males and 10.9 years in females. Epidemiological data on TTH in young subjects suggest prevalence rates of between 10 and 24% [9]. The earliest publications on CH in childhood date back to the 1970s [28,29] and in 1980, an interesting description of cluster symptoms in an infant was published [30]. The past decade has seen few published reports on the outcome of childhood CH. A study by Lampl et al. [31] found childhood onset (11–18 years) of CH to have a prevalence of 0-1%.

Paroxysmal hemicrania (PH) is a rare headache with a prevalence of 0.02% [32]. Relatively few pediatric cases have been reported in the literature. Children as young as 3 years of age have reportedly been diagnosed with the disorder [33-35]. Similarly, SUNCT (short-lasting unilateral neuralgiform headache attacks with conjunctival injection and tearing) is an extremely rare disorder in children with very few cases reported in the literature [36]. No data are available about the evolution of these disorders following their observation in children.

### Evolution of the temporal pattern of migraine and tension-type headache over time (Table 1)

**Table 1:** Summary of the evolution of the temporal pattern of headache in children (M = migraine, TTH = tension-type headache; CH = cluster headache)

**Authors (year)**	**Type of study**	**Ages**	**Headache type**	**Headache evolution**	**Other symptoms, findings**
Guidetti et al. [15]	prospective, 8-year follow-up	age 12–26 yrs., (mean age 17.9 yrs.)	M, TTH	- 26.5% of the patients with M changed to TTH.	- high rate of headache remission in males.
				- 8.3% with TTH changed to M.	
				- 45% showed improvement, 34% were headache free, 15% unchanged, 6% worsened.	
Hernandez-Latorre et al. [38]	10-year prospective longitudinal study	>6 ≥ 10 yrs.	M	- favorable evolution among children with headache started after 6 yrs.	
				- 88% favorable clinical course; 12% placed on prophylactic treatment.	
Brna et al. [39]	prospective, 20-year follow-up	mean age 11.1 yrs.	M, TTH	- 66% improvement	- triggers of headache: stress, sleep deprivation, bright light, certain foods.
				- more TTH remission	- 38% motion sickness
				- 45% with mild headaches were headache free at 20 years;	- 13% rushes sensory disturbance
				- 18% with moderate/severe headaches were headache free at 20 yrs	- 7% Alice in Wonderland syndrome
				- 72% with moderate/severe headaches continued to have moderate or severe headaches at 20 yrs.	
Balottin et al. [40]	prospective, 4.2-year follow-up	< 6 yrs.	M, TTH	- headache persistence in the minority of cases associated with detection of somatic and psychiatric disorders.	-
Kienbacher et al. [12]	prospective	17.6 ± 3.1 yrs.	M, TTH	- 25.7% were headache free, 48.6% still M and 25.7% still TTH at the follow-up.	- unfavorable outcome: longer time between headache onset and first consultation
					- good prognosis: changing headache location at baseline and long clinical follow-up
Kelman et al. [42]	cross- sectional study, retrospective analysis	mean age 37.7 ± 11.7 yrs	M	- new headache triggers: hormones, alcohol, smoking, neck pain;	- stress as a trigger, photophobia, phonophobia and dizziness, decrease with age;
				- shift in headache location toward the neck.	- decrease in the strength of attacks, and reduced need to sleep or rest during headache
				- increase in rhinorrhea and lacrimation	
Virtanen et al. [43]	prospective, controlled study	6-13 yrs	M, TTH	- 1/2 of M unchanged at 6 yrs; 32% changed to TTH.	- osmophobia, dizziness and balance disturbances became more typical with age
				- TTH unchanged in 35%; 38% changed to M.	- restlessness, flushing and abdominal symptoms became less marked.
				- at preschool age the location of headache was bilateral and	
				- supraorbital; at puberty bilateral and temporal.	
Gaßmann et al. [44]	prospective, 4-year longitudinal study	8-15 yrs	M, TTH	- M more frequent in girls than boys, and this difference increased significantly with age.	
				- TTH dropped from 57% among 8-year-olds to 45.6% among 15-year-olds.	
				- M increased with age from 10 to 17.1%.	
Slater et al. [45]	prospective, retrospective	mean age 11.7 ± 3.6	M	- early onset of headache in boys	- higher levels of disability as shown by PedMIDAS and no. of missed schooldays in girls.
				- boys’ headache: squeezing at the top of the head; sharp pain at the back of the head	- older children were more disabled.
				- girls reported frontal and temporal headache, and pain in the back of the head; pain: throbbing, pressure, constant and sharp.	
				- girls experienced more frequent and longer mean duration of headaches.	
				- older children reported greater headache frequency.	
Ozge et al. [46]	prospective, longitudinal, school-based six-year interval analysis	8-18 5562 children of whom 1155 followed up as adolescents	M, TTH	- childhood headache persists in adolescence, although the diagnoses mostly (71.3%) changed over time.	- PedMIDAS score higher in subjects with parents with headache history
				- M prevalence increased from 10.4% in childhood to 18.6% to adolescence in the same study sample after a 6-year interval.	- No supportive correlation with BMI MOH frequency 13.0% with migraine and high PedMIDAS score predominance
				- Headache prevalence increased with advancing age, especially in females; stress factors were the most important determinants.	
				- M negatively affects daily living activities in adolescents.	
Wöber- Bingol et al. [47]	cross- sectional study, retrospective analysis	3- 69 yrs	M with or without aura	**-** decrease in headache prevalence from childhood to adulthood in males	- aggravation by physical activity found to be decreased with age.
				- increasing of headache duration with age, prevalence of unilateral and pulsating pain, photo and phonophobia in girls.	- aura more frequent among ages 15–40 years
					- no gender differences in aura symptoms.
Maytal et al. [10]	retrospective	18 yrs or younger	CH	- clinical features of CH in childhood similar to those in adults.	-changes in associated symptoms over the years in a small number of patients
				- increase in frequency and duration of cluster periods with age in 40%.	
				- decrease in duration of cluster periods in 6%	
				- short cluster periods in 23% of patients in childhood and in 6% of patients in adulthood.	
				- CH shifted sides in one patient.	
Lampl [31]	epidemiological + 1 case report	7 years	CH	- no data about evolution of headache	**-** no differences between childhood and adolescent CH with regard to type of pain, associated symptoms and predominance in males.
				- frequency and duration may increase or remain unchanged over time if pts are untreated	
				- although brief remissions may occur, spontaneous resolution of CH is rare.	
Antonaci et al., [48]	case report and literature review	case of an 11-year-old boy	CH	- first bout 8 months; second bout 2 months, with the same pain characteristics.	
				- this patient as a ‘variant’clinical picture	
Arruda et al. [49]	prospective case report	9, 12 and 13 yrs	CH	- no differences between childhood and adult CH regarding frequency and duration	
				- good response to indomethacin in two cases;	
				- sustained long-term medical and/or spontaneous remission occurred in two patients.	

Guidetti and Galli [15,37] conducted a long-term prospective study in which 100 subjects (60 females, 40 males aged 12–26 years with a mean age of 17.9 years) were examined in 1988 and then re-evaluated in 1996, in order to ascertain the evolution of migraine and TTH after an eight-year interval. Of the 100 patients seen at the eight-year follow-up, 45% showed an improvement and 34% were headache free, whereas the clinical pattern was unchanged in 15%. The remaining 6% presented a worsening of headache. Migraine showed a lower tendency to remit compared with TTH (28.1% vs 44.4%). Headache prognosis was found to be mostly gender related, with the males showing a higher rate of headache remission than the females (52.5% vs 21.7%). It was concluded that the characteristics early-onset headache tend to change over time, and that early-onset forms have a high tendency to remit or to improve (15).

Hernandez-Latorre et al. [38], in a 10-year prospective longitudinal study of 181 migrainous children, evaluated the possible existence of markers of favorable and unfavorable evolution. In 24% of the children, the onset of migraine had occurred before the age of 6 years, while in 57% it had occurred between 6 and 10 years of age. It was concluded that the proportion of patients whose headache showed a favorable evolution was higher among those whose symptoms appeared after the age of 6 years than among those in whom the disease started earlier. Children with headache beginning before the age of 6 were found to have a 4.2 times greater risk of an unfavorable evolution than those whose symptoms appeared between 6 and 10 years of age. Eighty-eight percent of the patients showed a favorable clinical course, while 12% had to be placed on prophylactic treatment due to increase of their headaches.

Brna and coworkers [39] also conducted a long-term prospective study evaluating the prognosis of childhood headache. Ninety-five percent of their series of headache patients were first studied in 1983 and interviewed after 10 and 20 years (mean follow-up 20.1 ± 0.4 years). The mean age at diagnosis was 11.1 ± 3.0 years, with a male-to-female ratio of 1:4. Improvement of headache was found in 66% of the patients included in this study and TTH was found to be more likely than migraine to remit. Headache severity at diagnosis was predictive of headache outcome at 20 years. Of the patients with mild headache at diagnosis, nine (45%) were headache free at 20 years; of those with moderate or severe headache at diagnosis, seven (18%) were headache free 20 years later while the other 29 (72%) were still affected by moderate or severe headache. The provoking factors most commonly reported at 20-year follow-up were: stress (32%), sleep deprivation (13%), bright light (13%), and certain foods (10%). Associated headache symptoms were common in patients with migraine and TTH. Thirty-eight percent reported motion sickness at 20-year follow-up vs 23% at 10 years. Only one patient had somnambulism at the 10-year follow-up. The rushes sensory disturbance was reported in 13% at 20 years and in 17% at 10 years, whereas the Alice in Wonderland syndrome occurred in 7% at 20 years and in 10% at 10 years.

As regards the evolution of early-onset idiopathic headache over time, our group published a prospective evaluation of 25 headache patients referred before the age of 6 years and evaluated through a long-term clinical follow up (mean duration 4.2 years) [40]. In this study headache diagnosis was based not only on the HIS 1988 and ICHD-II criteria [41], but also on “alternative” clinical criteria: duration < 1 hour in migraine without aura and < 30 minutes in TTH. The influence of early somatic disorders, “life events”, and psychiatric disorders on the onset and course of headache were investigated. At the end of follow-up headache persistence was associated with early detection of somatic disorders and the presence of psychiatric disorders. No significant role was found for gender, history of headache in family members, headache diagnosis at onset or psychiatric comorbidity at first headache center visit.

Another long-term prospective study was published in 2006 by Kienbacher et al. [12]. These authors evaluated children and adolescents with migraine and TTH, in order to investigate the evolution, over time, of their clinical features and headache diagnosis. The study included 227 patients (52.5% female, 47.6% male) approximately 6.6 years after their first consultation at a headache center. Of the 140 patients initially diagnosed with migraine, 25.7% were headache free, 48.6% still had migraine, and 25.7% had TTH at the end of the follow-up. Of the 87 patients initially diagnosed with TTH, 37.9% were headache free, 41.4% still had TTH, and 20.7% had migraine. Consequently, it was shown that 30% of children and adolescents consulting a headache center because of migraine or TTH become headache free in the long term, while 20-25% switch from migraine to TTH or vice versa. Female gender, and a longer time between headache onset and first headache center visit tended to predict an unfavorable outcome; while changing headache location at baseline and long clinical follow-up of headache seemed to be favorable prognostic factors. Of the 34 patients with chronic daily headache, including eight with chronic migraine, four with probable chronic migraine, 15 with chronic TTH and seven with probable chronic TTH, 31 (91%) had a favorable outcome: 12 became headache free, 14 had headache on ≤4 days per month, and five patients had headache on 5–12 days.

Kelman and coworkers [42] evaluated the influence of age on migraine features in a cross-sectional study of 1009 patients, divided into three age groups: 16–29 years, 30–49 years, and 50 years and over. They found that stress (as a trigger), photophobia, phonophobia and dizziness (as accompanying symptoms) declined with age; moreover, the severity of headache and the need to sleep or rest during attacks also decreased with age. In some cases, increasing age introduced new headache triggers, such as hormones (this factor peaked in women aged 30–49 years), alcohol, smoking and neck pain, while the location of the headache shifted towards the neck. Another change with age was an increase in certain symptoms associated with headache: rhinorrhea and lacrimation.

Virtanen et al. [43] in a prospective study evaluated changes in headache from preschool age to puberty. Finnish families (n = 1132) with a 6-year-old child were questioned. Children affected by headache in the previous six months and their controls were clinically examined at the age of 6 and followed up at the age 13 years. During the follow-up, half of the headaches originally classified as migraine at age 6 years remained unchanged while 32% turned into TTH. In the children with TTH at 6 years, the situation remained unchanged in 35%, while 38% switched to migraine. The most common headache locations were bilateral and supraorbital at preschool age and bilateral and temporal at puberty. During the follow-up, symptoms accompanying headache, such as osmophobia, dizziness and balance disturbances, became more typical, whereas restlessness, flushing and abdominal symptoms became less marked.

In a four-year longitudinal study of pediatric headache in children and adolescents aged 7–14 years, Gassmann et al. [44] analyzed headache prevalence and evolution. The sample consisted of 8800 households with a child aged between 7 and 14 years. A total of 4159 households responded in both year 1 and year 2, yielding 3984 valid parent questionnaires. In more than half of the children and adolescents with headache (57.0%), the frequency of head pain remained stable over the one-year period, while improved and worsened frequency of occurrence were found in 22.3% and 20.7% of subjects, respectively. Girls experienced migraine more often than boys, and this difference increased significantly with age. At the age of 15 years, the prevalence of migraine in girls was nearly twice that recorded in boys. The proportion of children with TTH decreased with age, from 57% in 8-year-olds to 45.6% in 15-year-olds. The proportion of subjects with migraine increased with age from 10 to 17.1%.

Slater et al. [45] performed a prospective study of 4121 pediatric headache patients. They assessed headache characteristics at an initial consultation and subsequently re-examined the patients at five follow-up visits. A retrospective analysis of the data was completed considering all the patients who completed the questionnaires between January 1998 and October 2007. Fifty-eight percent of the sample were females. Several gender-related differences in the pediatric headache profile were found: the boys began to have headache earlier (mean age: 10 yrs) than the girls (mean age: 12.5 yrs); the boys more frequently described “squeezing” headache pain and more frequently reported pain located in the “top of the head” and sharp headache pain located at the back of the head. The girls were more likely to report frontal pain, temporal, pain and pain in the back of the head. They also more frequently described their headache pain as “throbbing”, “pressure”, “constant” and “sharp”. The girls experienced more frequent attacks and headaches with a longer mean duration compared with the boys. Older children reported greater headache frequency than did younger children. Girls showed higher levels of disability than boys considering both PedMIDAS score and number of school days missed. The older a child was at the initial visit, the higher the PedMIDAS score, meaning that the older children were more disabled by headache. According to the ICHD-II criteria (42), the majority of these patients had a diagnosis of migraine (91.1%), mostly without aura (78%). Fourteen percent had chronic migraine. Non-primary headaches were found in 1.6%, with 1.1% of these being post- traumatic.

In a broad, school-based, epidemiological follow-up study, Ozge et al. evaluated the natural history of migraine and TTH in children and adolescents using a validated headache questionnaire filled in under the supervision of a neurologist [46]. The initial sample of this study comprised 5562 children (10.4% migraine, 24.0% TTH); after an interval of six years, 1155 of these subjects subsequently studied as adolescents (18.6% migraine, 57.5% TTH). The main finding after the six-year interval was a high rate (71.3%) of variations in primary headache diagnosis with increasing age. Only 14.2% of the TTH and 16.5% of the migraine subjects were headache free at follow up. However, 56.6% of the sample had a new TTH diagnosis and 16.9% a new migraine diagnosis compared to childhood. In total, only 25.3% of the Turkish schoolchildren considered in this study became headache free. This study highlights the importance of changing and disturbing aspects of primary headache diagnoses with increasing age of the same patient group contributing to associate different headache phenotype.

Wöber-Bingöl et al. [47], in a cross-sectional study, examined 260 patients aged between 3 and 69 years. The case series included 169 females and 91 males suffering from migraine with or without aura. The prevalence of headache in the males was found to decrease markedly from childhood to adulthood. In the females, headache duration and the prevalence of unilateral, pulsating pain, photophobia and phonophobia increased. On the other hand, the prevalence of aggravation by physical activity decreased with age. Age-related differences in the clinical features of migraine were observed in the females, but could not be demonstrated in the males due to the relatively small number of men with migraine. Aura symptoms were found most often in the subjects aged 15–40 years and less often in the younger and older patients; the prevalence of aura symptoms did not differ between the males and females.

### Evolution of the temporal pattern of cluster headache over time

Both CH and the trigeminal autonomic cephalalgias are characterized clinically by repetitive attacks of intense headache accompanied by prominent cranial parasympathetic signs and symptoms. There are very few studies in the literature on the evolution of childhood CH. Maytal et al. [10], in a retrospective study, evaluated 35 patients with episodic and chronic CH arising at the age of 18 years or younger, including seven patients with onset prior to the age of 10. The male-to-female ratio was 6:1. The patients were followed up for 18 years, and the clinical features of CH attacks in childhood were found to be similar to those that typically occur in adulthood. The frequency and duration of cluster periods and the frequency of single headache attacks during cluster periods increased in 14 subjects (40%). Only two patients (6%) reported a decrease in the duration of cluster periods. Very short cluster periods, of two weeks or less, occurred in 23% of patients during childhood and in 6% during adulthood. On comparison of cluster period duration in childhood versus adulthood in individual patients, 13 (37%) patients showed longer-lasting cluster periods in adulthood, while only two (6%) patients showed a decrease in the duration of cluster periods over time. A small number of patients noted changes in associated symptoms over the years. Of these, only six reported more symptoms in adulthood than in childhood and only one patient’s symptoms shifted sides.

As regards the prevalence of CH in children, Lampl’s epidemiological study [31] showed that pediatric CH is rare and that the prevalence of childhood onset (11–18 years) is approximately 0.1%. The case of a 7-year old female who fulfilled the IHS diagnostic criteria for episodic CH was reviewed, but the paper in question did not provide data about evolution of the headache [31]. No differences in type of pain, associated symptoms and gender predominance (male) were found between childhood-onset CH and CH with onset in adolescence. When CH was left untreated the frequency and duration could increase or remain unchanged over time. Remissions, albeit brief ones, could occur, but spontaneous resolution of CH was rare.

According to our experience [48], CH has a prevalence of 0.09-0.1% in pediatric age, and the sex ratio is in favor of males (M/F: 3.2:1), albeit showing wide variation (1:1–6:1) (31, 47). Onset may occur as early as 3 years of age, although the number of cases with onset <10 years is relatively low. An atypical temporal pattern of episodic CH attacks was reported in an 11-year-old boy followed up for only nine months [48]. The first bout lasted eight months, and the second, in which the pain had the same characteristics, approximately two months. This patient could represent a ‘variant’ clinical picture.

In another prospective case report by Arruda et al. [49], three cases of CH in children and adolescents were discussed. Two of the patients had episodic CH (according to the ICHD-II criteria) and the other, affected by Down syndrome, had chronic unremitting CH. In this study, the frequency and duration of the attacks reported in childhood were similar to those that have been reported in adults, both for episodic and chronic CH. In two cases, a good response to indomethacin was observed, which makes the diagnosis questionable; moreover, sustained long-term medical and/or spontaneous remission occurred in two of the three patients.

## Discussion

This review suggests that there is still a paucity of literature data on the evolution and prognosis of headache starting in childhood and adolescence.

The present review concerned studies conducted after the publication of the IHS (1988) and ICHD-II criteria [17,41]. Current studies in this field may have some limits due to the fact that the ICHD-II diagnostic criteria for migraine and TTH are excessively restrictive for young children (children may have a short migraine duration, bilateral presentation of migraine and they also present a wide variability of associated symptoms). Many children, in fact, fail to meet all the ICHD-II criteria and a diagnosis of probable migraine often has to be made; therefore, improved diagnostic criteria are needed [36].

Another possible limit is the difficulty in designing studies that consider the transition from childhood to adulthood, given relatively short follow-up periods involved. Moreover, the possible effect of hormonal changes in puberty on the changing phenotype of primary headache disorders (especially migraine) with increasing age might be not independent of other environmental factors, such as stress, family variables, comorbidities, and so on.

However, these studies have shown that while the reported frequency of migraine in children varies slightly from country to country and region to region, headache overall is a common phenomenon [37]. Moreover, all the analyzed studies were methodologically rigorous, in most cases using a retrospective study design.

The discrepancies found between some studies may reasonably be attributed to several factors: differences in the number of headache patients enrolled, in the patient selection criteria – various populations were studied and different age ranges –, and in the sources of information used (parents versus child). In the past, a tendency toward parental underreporting of children’s headache has been reported. In an epidemiological study of 3461 parent reports of children aged between 7 and 14 years, a high correlation was found between the children’s and the parents’ reports of children’s headache frequency, but the correlations were much lower when other features were considered, with the parents’ perception of depression and anxiety found to be the least reliable variable [50]. In addition, very young patients constitute a particular diagnostic challenge due to their limited verbal and language skills (which make it difficult to establish the site of pain and its quality) and also to the fact that headache in very young children is often a non-specific complaint, associated with other illnesses.

There is a large consensus [51] that the natural temporal pattern of migraine may change over time: as attack frequency increases, the number of migraine features diminishes during the transformation period. At variance with what is observed in adults, adolescents with chronic daily headache have a temporal pattern with more migraine days than TTH [52]. It has also been shown that with increasing age, there is a decrease in the frequency of variables such as stress as a trigger, photophobia, phonophobia, dizziness, throbbing, pressure, stabbing, and being forced to sleep or rest during headache attacks. Factors that become more important with age are alcohol, smoking and neck pain as triggers, location of pain in the neck, and rhinorrhea/lacrimation [42]. Pediatric cases are often diagnosed as childhood periodic syndromes because children might not focus on their head pain, but rather on abdominal symptoms or vertigo. As they grow older their ability to describe their head pain improves and the description of the clinical picture of migraine becomes more detailed and accurate [53]. Pediatric migraine is more often bilateral, frontal and temporal than unilateral (35% vs 60% in adults). The duration of the headache is often much shorter – it can last as little as 30 minutes – and there is a higher incidence of light or noise sensitivity than in adults [54].

At present it can, to the best of our knowledge, only be speculated that several factors contribute to the development of this pathophysiological trait. Hormonal changes, different levels of stress exposure, and the life events and circumstances behind each stress exposure may be only some of the factors contributing to a changing migraine phenotype over time.

Certain primary headaches may remit in a certain period, but the remission rate of headaches at short-term follow-up cannot be considered indicative of a resolution pattern in the absence of parallel, long-term studies. When patients were followed up for 10 years or less, headaches were shown to improve or remit in 60-80% of cases [2]. Persistence of primary headache at 10-year follow-up appears to be more predictive of headache persistence into adulthood, particularly if the primary headache is migraine (40). Guidetti et al. [37] found that migraine showed a lower tendency than TTH to remit. Accordingly, Bille et al. [2] found that 23% of patients were migraine free at 23 years of age, while more than 50% continued to have migraines at the age of 50 years. The prognosis of headache in children is negatively affected if the initial diagnosis is migraine and they have a changing headache location. A negative outcome has been correlated with a longer time between headache onset and first medical consultation [54].

As regards the impact of sex hormones on migraine in women [55,56], some authors have analyzed [57] whether the onset of menarche affects the risk of developing migraine and headache frequency. An increased risk of headache was found in girls who had experienced menarche during the previous two years compared with girls yet to reach menarche. Aegidius et al. [58] examined the relationship between age at menarche and headache prevalence. The prevalence of headache was higher in girls with menarche at 12 years or younger than in those with menarche after the age of 12 years. In addition, they found a statistically significant trend toward decreasing prevalence of headache, migraine and TTH with increasing age at menarche. It was hypothesized that the inverse relationship between age at menarche and the prevalence of headache may be explained by a common pathogenetic factor such as sensitivity to estrogens, or by an increased susceptibility to headache due to early menarche. Hormonal changes as a migraine trigger were mainly observed in 30–50 year olds, and less in younger or older patients [42]. Hormonal changes might affect the pathophysiology of migraine in pediatric as in adult migraine and could partly explain the shift in predominance to girls during adolescence. Hormonal sensitivity is thought to be the basis for menstrual migraine [59,60].

Genotype may also play a role in the complex picture of headache evolution. An early onset of disease probably reflects increased biological predisposition or increased susceptibility to specific environmental risk factors [57]. The high incidence of family antecedents in migraineurs has been used as an argument to support the existence of genetic factors in the etiopathogenesis of this type of headache [59,61]. The incidence of family antecedents has been found to be significantly higher in children who first presented symptoms at a younger age [37]. It is very likely that a greater genetic load plays an important role in earlier onset of migraine and may determine a worse prognosis. Nevertheless, this reasoning needs to be backed up by statistical data.

There remain open issues that the available literature fails to address, such as the question of drug response (persistence/change/absence of response) at different ages, both in symptomatic and prophylactic regimens, and of the placebo response and the question of medication overuse headache [62] in childhood. Other issues still to be clarified in pediatric subjects are the evolution of the aura phase during the natural history of migraine and the evolution of more than one type of headache in a single patient.

## Conclusions

As shown by this systematic review, description of the evolution of headache from childhood to adulthood has been a focus of interest for several authors. The studies performed after the publication of the first IHS classification include both prospective and retrospective ones, the former being more reliable for defining the temporal pattern of headache. The availability of data about the evolution of primary headaches over a period of time is important, both from a clinical and a public health perspective. Indeed, the identification of prognostic factors of headache evolution over time should be an objective of future headache research to allow the development of prevention strategies. Headache may have a different incidence at different ages; a transition from migraine to other forms of headache or vice versa has been described; the clinical picture of migraine may change with age in some patients. However, we still lack sufficient data to assess whether these changes are hereditary or casual, and whether early abortive and prophylactic treatments play a role in the clinical shifts or in improvements of the temporal pattern.

As regards migraine, attacks in children are shorter lasting and present peculiar characteristics. The transition from childhood to adulthood brings a global lessening of the features of migraine is and a lower prevalence of migraine is observed in older patients. Approximately one in four migraine patients switched to TTH. Moreover, male gender is associated with remission, while early developmental disorders are associated with persistence of migraine. With increasing age, a decline in the frequency of variables such as stress as a trigger, and other pain characteristics or accompanying factors, was observed.

TTH is more likely than migraine to improve in the transition from childhood/adolescence to young adulthood. A switch to migraine was bserved in 25% of patients. As in migraine, male gender was found to be associated with remission and early developmental disorders to be associated with persistence.

CH is the type of headache on which least information is available, due to the relatively limited number of children affected. Episodic CH is more frequent than the chronic form. The frequency and duration of CH attacks in childhood are similar to those reported in adults. In the only retrospective study available, the frequency and duration of cluster periods and the frequency of single headache attacks during the bouts increased in less than 50% of patients as they approached adulthood.

More prospective studies following the evolution of headache into adulthood are required in order to achieve a deeper awareness of the different phenotypes of headache.

## Competing interests

The authors declare that they have no competing interests.

## Authors’ contributions

FA, CVI, reviewed the literature and drafted the manuscript ADS, reviewed the literature FG, AO,UB reviewed the manuscript and provided useful advice. All authors read and approved the final manuscript.
